# 2476: Identifying strangulated small bowel obstruction with machine learning

**DOI:** 10.1017/cts.2017.79

**Published:** 2018-05-10

**Authors:** Samuel David Zetumer, Hobart Harris

## Abstract

OBJECTIVES/SPECIFIC AIMS: Historically, logistic regression algorithms (LRAs) have failed to differentiate strangulated small bowel obstructions (SBOs) from nonstrangulated SBOs. Our hypothesis is that a machine learning algorithm (MLA) can differentiate strangulated from simple SBOs better than an LRA can. METHODS/STUDY POPULATION: We used records of patients presenting with acute SBO and managed with exploratory laparotomy to test and train algorithms. We compared MLA to LRA via area under the receiver operating characteristic curve (AUROC) and cut-off points maximizing sensitivity and specificity. RESULTS/ANTICIPATED RESULTS: With 192 patient records, the AUROC of the MLA was 0.85. At the sensitivity cutoff, the MLA had 100% sensitivity and 55% specificity. At the specificity cutoff, the MLA had 45% sensitivity and 100% specificity. We anticipate improvements as more records are incorporated, and that LRA will underperform MLA across all measures. DISCUSSION/SIGNIFICANCE OF IMPACT: Our MLA represents a significant improvement over past LRAs, and may provide decision assistance to surgeons managing SBO. If this MLA maintains its high sensitivity, it may be used in the future to prevent unnecessary surgeries.

